# Quantitative proteomic characterization of human sperm cryopreservation: using data-independent acquisition mass spectrometry

**DOI:** 10.1186/s12894-019-0565-2

**Published:** 2019-12-16

**Authors:** Longlong Fu, Qi An, Kaishu Zhang, Ying Liu, Yue Tong, Jianfeng Xu, Fang Zhou, Xiaowei Wang, Ying Guo, Wenhong Lu, Xiaowei Liang, Yiqun Gu

**Affiliations:** 1 National Health Commission Key Laboratory of Male Reproductive Health, National Research Institute for Family Planning, Beijing, 100081 China; 2 Department of Male Clinical Research/Human sperm bank, National Research Institute for Family Planning & WHO Collaborating Center for Research in Human Reproduction, Beijing, 100081 China; 3 Graduate School of Peking Union Medical College, Beijing, 100730 China; 4 Department of Reproductive Medicine, The Afliated Hospital of Qingdao University, Qingdao, Shandong 266000 People’s Republic of China; 5 Institute of Pediatric Research, Children’s Hospital of Soochow University, Suzhou, Jiangsu Province, 215025 China

**Keywords:** Cryopreservation, Fertility preservation, Reproductive techniques, assisted, Metabolic networks and pathways

## Abstract

**Background:**

Human sperm cryopreservation is a simple and effective approach for male fertility preservation.

**Methods:**

To identify potential proteomic changes in this process, data-independent acquisition (DIA), a technology with high quantitative accuracy and highly reproducible proteomics, was used to quantitatively characterize the proteomics of human sperm cryopreservation.

**Results:**

A total of 174 significantly differential proteins were identified between fresh and cryoperservated sperm: 98 proteins decreased and 76 proteins increased in the cryopreservation group. Bioinformatic analysis revealed that metabolic pathways play an important role in cryopreservation, including: propanoate metabolism, glyoxylate and dicarboxylate metabolism, glycolysis/gluconeogenesis, and pyruvate metabolism. Four different proteins involved in glycolysis were identified by Western blotting: GPI, LDHB, ADH5, and PGAM1.

**Conclusions:**

Our work will provide valuable information for future investigations and pathological studies involving sperm cryopreservation.

## Background

Human sperm cryopreservation plays an important role in the treatment of male infertility when the male partner has severe abnormalities in semen parameters [1]. Furthermore, this technique is a simple and effective approach to male fertility preservation [2, 3], and is the only approach used in the clinic in many countries [4]. However, the cryopreservation protocols will induce a notable decrease in sperm motility, as well as affecting other parameters, including: membrane and acrosome integrity, DNA fragment, and reactive oxygen species [5, 6]. Certain intracellular and extracellular factors have been shown to lead to cryo-damage [7, 8]. However, the related pathogenesis of sperm cryo-damage during the process of cryopreservation shaould be clarified by further study.

Proteomics, especially sperm proteomics, is a new field in human reproduction studies. Ptoteomics can elucidate complex biological systems, including sperm motility and fertilization, and it can discover the potential pathogenic mechanisms and the biomarkers associated with male infertility [9, 10]. Data-independent acquisition (DIA) is, a novel proteomics technology, based on acquiring fragment ion information for all precursor ions within a certain range of m/z values [11]. Different from data-dependent acquisition (DDA), the DIA strategy has the characteristics of high quantitative accuracy and high reproducibility; and the experimental method has broad applicability: up to 5000 proteins can be detected and quantified in one experiment [11, 12]. To the best of our knowledge, this technique has not previously been applied to the study of human cryopreserved sperm.

For these reasons, the aim of the current study is to compare the proteomic differences between fresh and cryopreserved human sperm, using DIA mass data. A further understanding the sperm proteins would be very helpful in explaining sperm cryoinjury and may establish biomarkers for sperm motility.

## Methods

### Ethics statement and sample collection

The study was approved by National Research Institute for Family Planning Ethics Committee on Human Subjects (2018018). Ejaculates were obtained from healthy semen donors attending the Human Sperm Bank, National Research Institute for Family Planning, in Beijing China. Informed consent was obtained from all the sperm donors involved in the study.

### Study design and sample collection

Paired design was applied to this study. Every semen sample was divided into two parts: one for the cryopreserved group and the other for the fresh group. Our focus was the difference between the cryopreserved and fresh human sperm.

A total of 14 semen samples from 14 qualified healthy sperm donors were collected. The ages of the sperm donors were between 22 years and 29 years. One ejaculate was collected from these volunteers by masturbation after 3–5 days of sexual abstinence. Routine semen analyses were performed by Computer Aided Sperm Analysis (CASA)(HTM-IVOS, USA), according to the World Health Organization (WHO 2010) guidelines.

Among the whole sample of each group, 9 cryopreserved and fresh samples were used for proteomics analysis. Similar to the previous study [13], the 9 donors’ ejaculates (each one divided into fresh and cryopreserved vials) were divided into 3 groups of 3. Then 5 for Western blot analysis.

### Semen cryopreservation protocols

After complete liquefaction, 2 mL of each semen sample was divided into two parts: one for cryopreservation and the other placed in a 37 °C water bath as the control. Glycerol-egg-yolk-citrate (GEYC) was used as cryoprotectant containing 15% glycerol, 20% egg yolk, 1.3% glycine, 1.5% glucose, and 1.3% sodium citrate tribasic dehydrate, with a pH of 6.8–7.2. One volume of GEYC was added to two volumes of semen, drop by drop with swirling, and the mixture was incubated at 30–35 °C for 5 min. A slow sperm freezing method was performed according to the standardized programmable freezers (Kryo 360–1.7, Planner, United Kingdom) in our unit [12, 14]. Briefly, one volume of GEYC cryoprotectant was added to two volumes of semen, and the program was as follows: the samples tubes waerw cooled at 1.5 °C per minute from 20 °C to − 6 °C, at 6 °C per minute to − 100 °C, and at − 100 °C for 30 min, then the sample tubes were transferred to liquid nitrogen. After being preserved in the liquid nitrogen for a minimum of 2 days, a small portion (10 μL) of the frozen sample was thawed for sperm quality assessment.

### Protein extraction and digestion

Protein extraction and digestion were performed as described previously [15]. The fresh and cryopreserved semen were centrifuged at 800 g for 10 min to remove seminal plasma, round cells and the cryoprotectant. Then, the sperm were washed three times with phosphate-buffered saline (PBS). Five hundred microliters of lysis buffer [8 M UREA, 100 mM Tris-HCl, pH 7.6, 1 mM PMSF (phenylmethylsulfonyl fluoride)(Roche, Germany)] was added to each sample. Then, the samples were sonicated(Sonics, USA) at 20 joules for 2 s × 10 at intervals of 15 s, and centrifuged at 18000 g for 15 min, after which the supernatant was extracted. Quantification was performed using the BCA method. The protein sample was frozen at − 80 °C. An aliquot of 20 μg was used for each sample to build the mixed and pooled library. Six samples and the pooled samples were subjected to enzymatic hydrolysis using a FASP (filter-aided sample preparation) enzymatic method.

Then, protein digestion was performed using the FASP protocol. In brief, protein (200 μg) was diluted with 50 mM dithiothreitol and incubated for 40 min at 56 °C. Further operation was carried out using the ultrafiltration tube method. The ultrafiltration tube was placed in a collection tube, and the protein sample was added to the ultrafiltration tube and centrifuged at 12000 g for 15 min. One hundred microliters of urea buffer containing 50 mM iodoacetamide was added to the ultrafiltration tube and it was then incubated for 20 min in the dark. The sample was washed twice by adding 100 μL urea buffer and centrifugede at 12000 g for 10 min to remove irrelevant substances. Then, 80 μL of 50 mM trypsin in NH_4_HCO_3_ was added to ultrafiltration tube, and the protein-to-enzyme ratio was 50:1. The samples were incubated at 37 °C for 16 h, and the released peptides were collected through centrifugation and directly determined by the protein concentration detection mode in the Nano-Drop instrument.

### Spectral library generation

The samples (1 μg) were analyzed on an EASY-Nano-LC mass spectrometer (Thermo, USA). The peptides were separated using 0.1% formic acid, containing iRT standard peptide(Buffer A) and acetonitrile containing 0.1% formic acid(Buffer B), along a linear gradient from 3 to 32% at 300 nL/min for 120 min. The gradient of chromatographic separation was as follows: 3–7% buffer B for 0-3 min, 7–20% buffer B for 80 min,20–32% buffer B for 24 min, 32–90% buffer B for 1 min, and 100% buffer B for 120 min. For DDA, the source was operated by Orbitrap Fusion (Thermo Scientific, USA), at 2.1 kV. The DDA scheme included a full MS survey scan from m/z350 to m/z 1500 at a resolution of 60 k full-width half-maximum (FWHM)(at m/z 200) with automatic gain control(AGC) set to 4E5 (maximum injection time of 50 ms). The parameters of MS2 were as follows: 30 k FWHM (@ m/z 200), isolation window 1.6 Th, AGC set to 4e5 (maximum injection time of 50 ms). For, high-energy collision dissociation (HCD): MS2 Activation (collision energy: 35) was used, and dynamic exclusion was set to 40s.

For the generation of the spectral library, DDA data analysis was performed using Protein Discoverer 2.1 SP1(SEQUEST HT). The database is human proteins database from UniProt: “uniprot-organism-9606 + reviewed-yes.fasta,” and iRT peptide sequences were added to the database: (>Biognosys|iRT-Kit|Sequence_fusion LGGNEQVTRYILAGVENSKGTFIIDPGGVIRGTFIIDPAAVIRGAGSSEPVTGLDAKTPVISGGPYEYRVEATFGVDESNAKTPVITGAPYEYRDGLDAASYYAPVRADVTPADFSEWKLFLQFGAQGSSPFLK).

Raw data were analyzed according to the user guide of the software. The parameters were set as follows: the initial mass tolerance for precursor ions was: 10 ppm, and the mass tolerance for product ion spectra was 0.02 Da. Tryptic cleavage was selected, and the maximum allowable number of missing cleavage was 2. Carbamidomethyl of cysteine was set as the fixed modification, while oxidation of methionine and N terminal acetylation were set as variable modifiers. The identifications were filtered to satisfy an FDR of 1%, and the unique peptide number per protein was greater than one. Then, the result was used to build the DDA spectral library in Spectronaut Pulsar X(Biognosys, Switzerland). The library parameters used the default optimal parameter “GBS factory setting”.

### Protein identification and quantitation

For DIA, 2 μg peptides was taken from each sample and mixed with appropriate iRT standard peptides. Each sample was tested by DIA mass spectrometry for 2 h. The method consisted of a full MS1 scan at a resolution of 60 K from m/z 350 to m/z 1500, with AGC set to 4E5 (maximum injection time of 50 ms), followed by 46 DIA windows acquired at a resolution of 30 K FWHM with AGC set to 5e5 (maximum injection time of 55 ms); HCD: MS2 activation (collision energy: 35).

Finally, qualitative and quantitative analyses of DIA raw data were performed in Spectronaut Pulsar X. Database parameters were as follows: peptides FDR\ PSM FDR\ proteins FDR was 1%; at least three proteins were selected for each peptide, at most 6 optimal ion generation library spectra were selected, and iRT calibration R^2^ > 0.8. The quantitative parameters were set as follows: the iTR standard used a nonlinear fit (local (non-linear) regression), the protein identification used a precursor Qvalue cutoff 0.01, the protein Qvalue cutoff of 0.01, the protein quantification used the peak area of sub-ions, and the average Intensity of at least three sub-ions was selected to quantify the protein. Protein quantification uses the ion peak area, and at least the average intensity quantification of three sub-ions is selected. Paired t-test was used as the analytical approach and *P*-value of 0.05 was set.

### Bioinformatics analysis

All significantly differential proteins were used as input. The OmicsBean analysis tool [16] was used to retrieve the Gene Ontology Consortium categories, including: molecular function (MF), cellular component (CC) and biological process (BP). KEGG [17] pathway enrichment analysis was performed using the Kanehisa databases web service (https://www.kanehisa.jp/en/archive.html). The protein-protein interaction network (PPI) was constructed using the STRING [18] web service (http://www.string-db.org/).

### Western blotting

According to our previous experience [19], the sperm protein samples were separated by 10% SDS polyacrylamide gel electrophoresis. The samples were then transferred to a polyvinylidene fluoride membranes, blocked with 2% (w/v) skim milk for 1 h, and incubated overnight with the primary antibody of glucose-6-phosphate isomerase (GPI), lactate dehydrogenase B (LDHB), alcohol dehydrogenase 5 (ADH5), and phosphoglycerate mutase 1 (PGAM1) (1:1000) (Abclone, China) at 4 °C temperature. After three washes with TBST, the membranes were incubated with horseradish peroxidase (HRP) in combination with anti-IgG for 1 h at room temperature (18–22 °C). Enhanced chemiluminescence revealed immunoreactivity. The relative signal intensity of protein bands was analyzed with Quantity One v.4.6.2.

### Statistical analysis

Data are expressed as the mean ± SD and were analyzed with SPSS22.0 software (IBM, USA). Paired t-test was used as the analytical approach, and a *P*-value of 0.05 was set.

## Results

### Sperm motility

We aimed to assess the impact of cryopreservation on sperm motility (Table 1). Compared with fresh semen, a significant decrease in the percentage of progressive sperm, and sperm motion parameters was observed in cryoperserved sperm, including average path velocity (VAP), straight line velocity (VSL), and curvilinear velocity (VCL).

**Table 1:** Characteristics of motility of fresh and post-thaw human sperm (*n* = 14) (Mean ± SD)

	Percentage of PR(%)	VAP (μm/s)	VSL (μm/s)	VCL (μm/s)
Fresh sperm	67.0 ± 7.5	42.4 ± 0.9	31.0 ± 1.7	65.2 ± 1.2
Post-thaw sperm	45.3 ± 2.51*	37.8 ± 2.4*	29 ± 0.5*	58.6 ± 5.8*

*: *P* < 0.05 compared with the Fresh group

*PR* progressive; *VAP* average path velocity; *VSL* straight line velocity; *VCL* curvilinear velocity.

### Quantitative results of differential proteins

Compared with the spectral library, a total of 29,495 unique peptides and 5246 proteins were identified. Finally, 3790 proteins were quantitatively analyzed. Using the 1.5-fold or 0.67-fold change and the FDR-adjusted *p*-value of 0.05 as cutoffs, 174 (4.6%) significantly differential proteins were identified between fresh and cryopreserved sperm: 76 were increased and 98 were decreased in the cryopreserved group. The detail of the differential proteins due to cryopreservtaion are provided in the Additional file 1: Table S1.

### Gene ontology enrichment

**Fig. 1:**
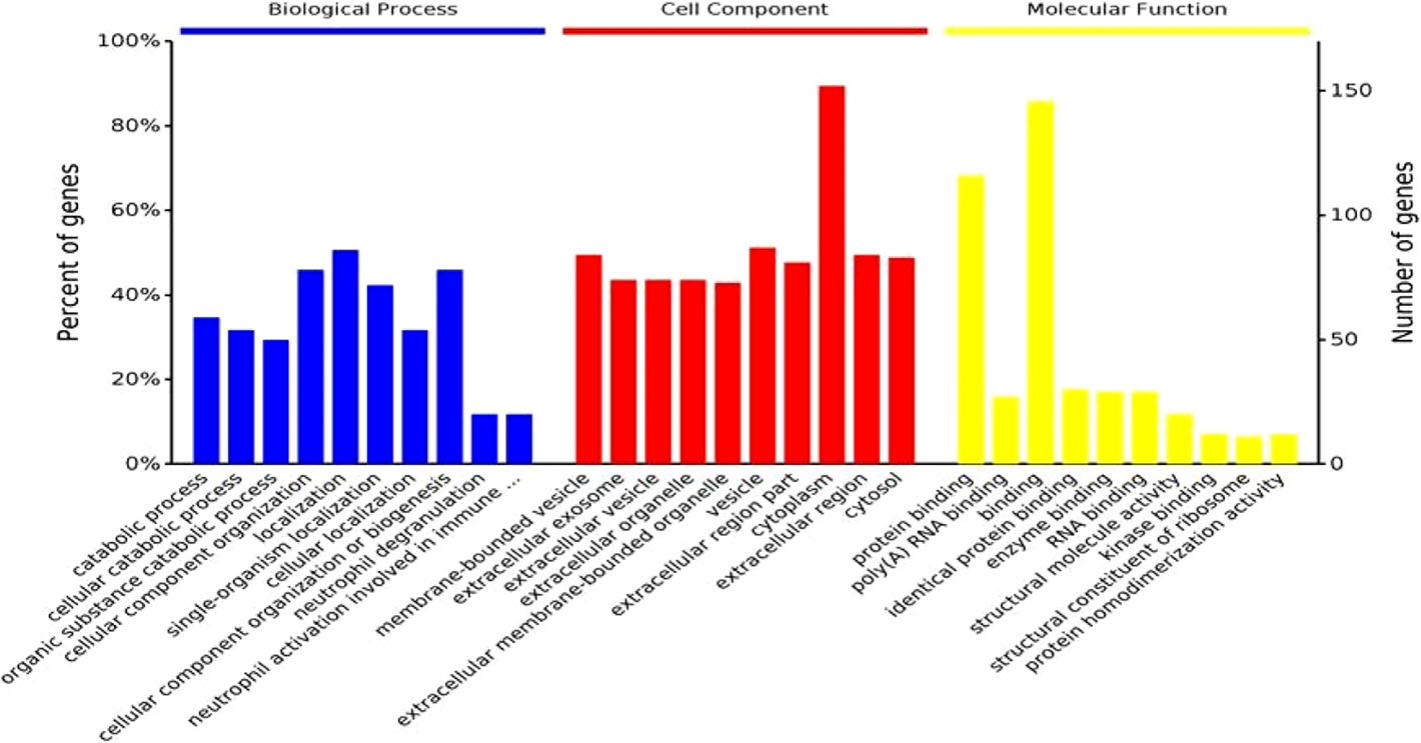
Gene Ontology analysis classification and the enrichment result of differentially expressed proteins in the human freeze-thaw samples compared to the fresh group, in terms of Biological Process, Cell Component and Molecular Function

Enrichment analysis was performed based on the 174 significantly deregulated proteins. A total of 1544 biological processes(BPs), 275 cell components(CCs) and 261 molecular functions(MFs) were involved in the GO enrichment, and there were significant differences between the fresh and cryopreserved sperm(*P* < 0.05). The number of differential proteins contained in each entry and its percentage of total differential protein are shown in Fig. 1. The detailed results of the GO enrichment are provided in Additional file 2: Table S2.

### KEGG pathway and protein-protein interaction (PPI) network analysis

**Fig. 2:**
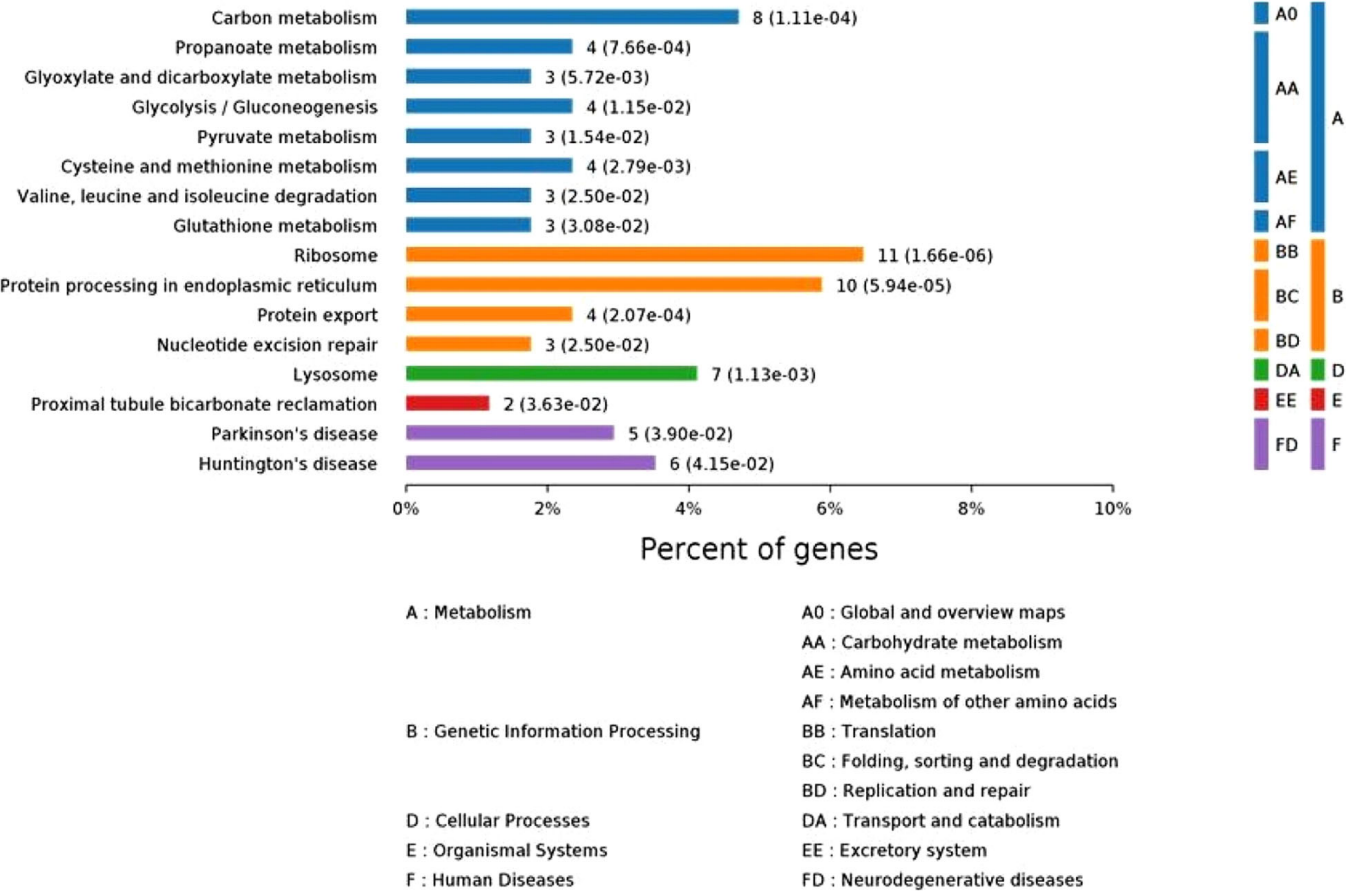
Kyoto Encyclopedia of Genes and Genomes(KEGG) pathway enrichment analysis

**Fig. 3:**
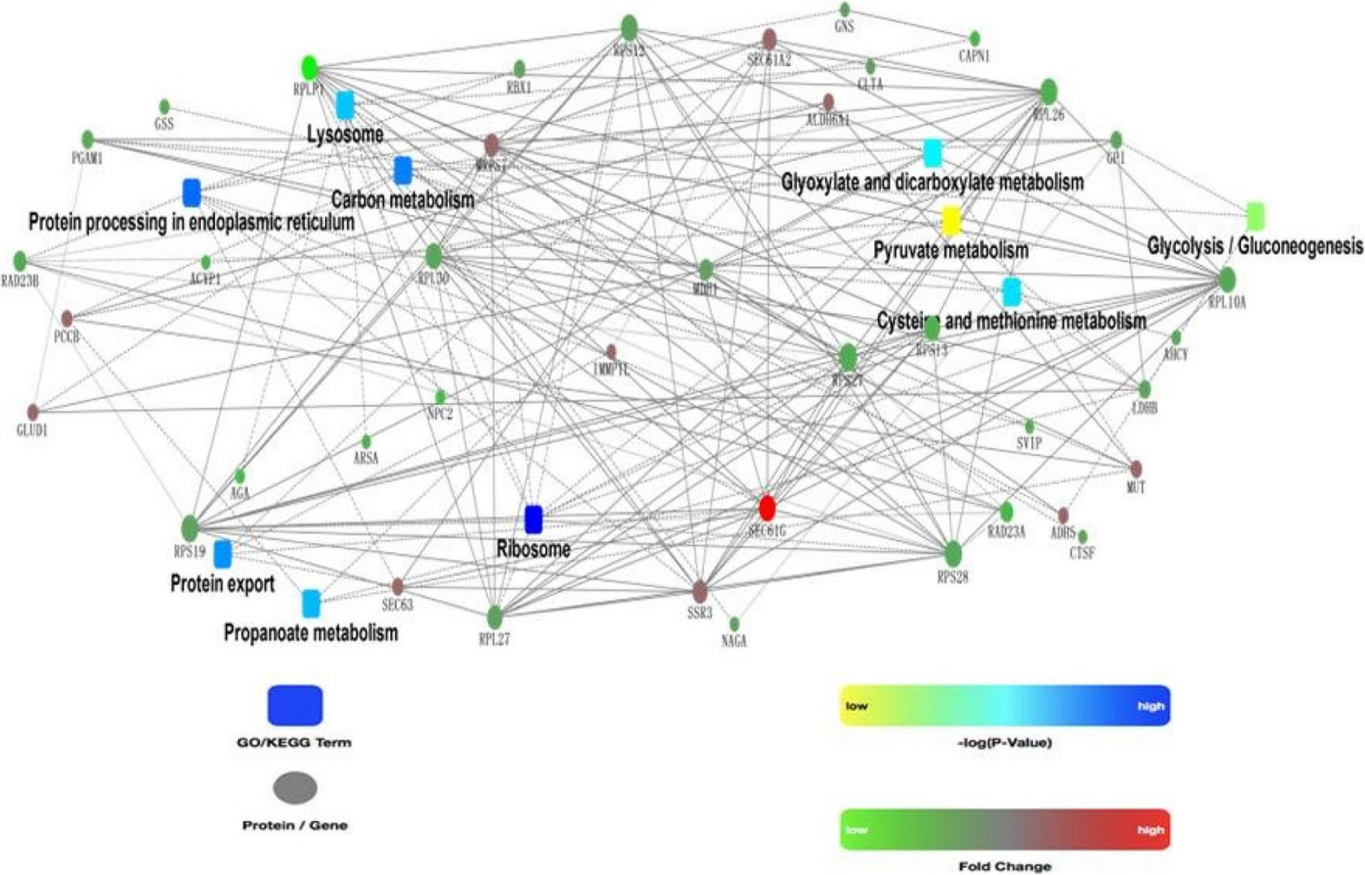
Protein interaction network diagram (STRING)

Pathway enrichment analysis was also performed by KEGG enrichment in order to identify the major biochemical pathways and signal transduction pathways. Several pathways were significantly disturbed in cryopreserved sperm, such as: ribosome, carbon metabolism and lysosome (Fig. 2). The details of the 16 pathways with significant differences are shown in Table 2. To show the main interactions and regulatory relationships of these proteins, the PPI network was constructed (Fig. 3).

**Table 2:** Detailed information of the Kyoto Encyclopedia of Genes and Genomes (KEGG) pathway enrichment results

Pathway Name	Pathway ID	*P* value	Genes: Name|Ratio(Cryopreservated/Fresh)	Count	Pop Hit	Class
Ribosome	hsa03010	1.66E-06	RPL10A|0.573336532743;RPS19|0.631553739412;RPS13|0.523487855699; RPL27|0.636693323276;RPS12|0.620311406135;RPS28|0.556462384813; MRPS7|1.61901451638;RPL30|0.585111111172;RPL26|0.525298518938; RPLP1|0.201692403302;RPS27|0.545857932715	11	138	Genetic Information Processing
Protein processing in endoplasmic reticulum	hsa04141	5.94E-05	CAPN1|0.483846314217;SEC63|1.64488179;NSFL1C|0.532234501825; RAD23B|0.544847606418;RAD23A|0.449570898437;SEC61A2|1.59938044965; SVIP|0.57175573788;SSR3|1.53947957741;SEC61G|12.6538069403; RBX1|0.63561326146	10	166	Genetic Information Processing
Carbon metabolism	hsa01200	1.11E-04	GPI|0.594470429855;MUT|1.64145141153;ALDH6A1|1.80998863199;MDH1|0.61379399419;ADH5|1.57385688652;GLUD1|1.60104289531;PGAM1|0.554594456772;PCCB|1.67972523554	8	113	Metabolism
Protein export	hsa03060	2.07E-04	SEC63|1.64488179;SEC61A2|1.59938044965;SEC61G|12.6538069403;IMMP1L|1.67051120115	4	23	Genetic Information Processing
Propanoate metabolism	hsa00640	7.66E-04	MUT|1.64145141153;ALDH6A1|1.80998863199;LDHB|0.606365006071;PCCB|1.67972523554	4	32	Metabolism
Lysosome	hsa04142	1.13E-03	CTSF|0.624627283152;GNS|0.637102261894;ARSA|0.494575557376;NAGA|0.650162388685;NPC2|0.426290016224;AGA|0.43725788109;CLTA|0.666776335796	7	123	Cellular Processes
Cysteine and methionine metabolism	hsa00270	2.79E-03	AHCY|0.597470127607;MDH1|0.61379399419;LDHB|0.606365006071;GSS|0.585813890146	4	45	Metabolism
Glyoxylate and dicarboxylate metabolism	hsa00630	5.72E-03	MUT|1.64145141153;MDH1|0.61379399419;PCCB|1.67972523554	3	28	Metabolism
Glycolysis / Gluconeogenesis	hsa00010	1.15E-02	GPI|0.594470429855;LDHB|0.606365006071;ADH5|1.57385688652;PGAM1|0.554594456772	4	67	Metabolism
Pyruvate metabolism	hsa00620	1.54E-02	MDH1|0.61379399419;LDHB|0.606365006071;ACYP1|0.490664299807	3	40	Metabolism
Valine, leucine and isoleucine degradation	hsa00280	2.50E-02	MUT|1.64145141153;ALDH6A1|1.80998863199;PCCB|1.67972523554	3	48	Metabolism
Nucleotide excision repair	hsa03420	2.50E-02	RAD23B|0.544847606418;RAD23A|0.449570898437;RBX1|0.63561326146	3	48	Genetic Information Processing
Glutathione metabolism	hsa00480	3.08E-02	GSS|0.585813890146;GGCT|0.573964558442;GSTK1|1.70055565858	3	52	Metabolism
Proximal tubule bicarbonate reclamation	hsa04964	3.63E-02	MDH1|0.61379399419;GLUD1|1.60104289531	2	23	Organismal Systems
Parkinson’s disease	hsa05012	3.90E-02	ATP5F1|1.571159587;VDAC3|1.63594176768;UCHL1|0.613051768866;NDUFB8|1.71896191444;MT-ND3|1.54131079277	5	142	Human Diseases
Huntington’s disease	hsa05016	4.15E-02	DCTN2|0.634124905506;ATP5F1|1.571159587;VDAC3|1.63594176768;CLTA|0.666776335796;AP2S1|4.07535354503;NDUFB8|1.71896191444	6	193	Human Diseases

The “Pop hit” is the total number of proteins in the pathway; the “Count “is the actually matched number

According to the KEGG analysis, the results showed found that the metabolic pathways play an important role in cryopreservation (Fig. 2), including: propanoate metabolism, glyoxylate and dicarboxylate metabolism, glycolysi/gluconeogenesis, and pyruvate metabolism. Most of these pathways were down regulated in the cryopreserved sperm group (the green in the Fig. 3).

### Validation of the glycolysis metabolic proteins

To further validate the outcome of the KEGG analysis, we used Western blotting to quantify the four dysregulated protein enzymes in glycolysis: GPI, LDHB, ADH5, and PGAM1.

**Fig. 4:**
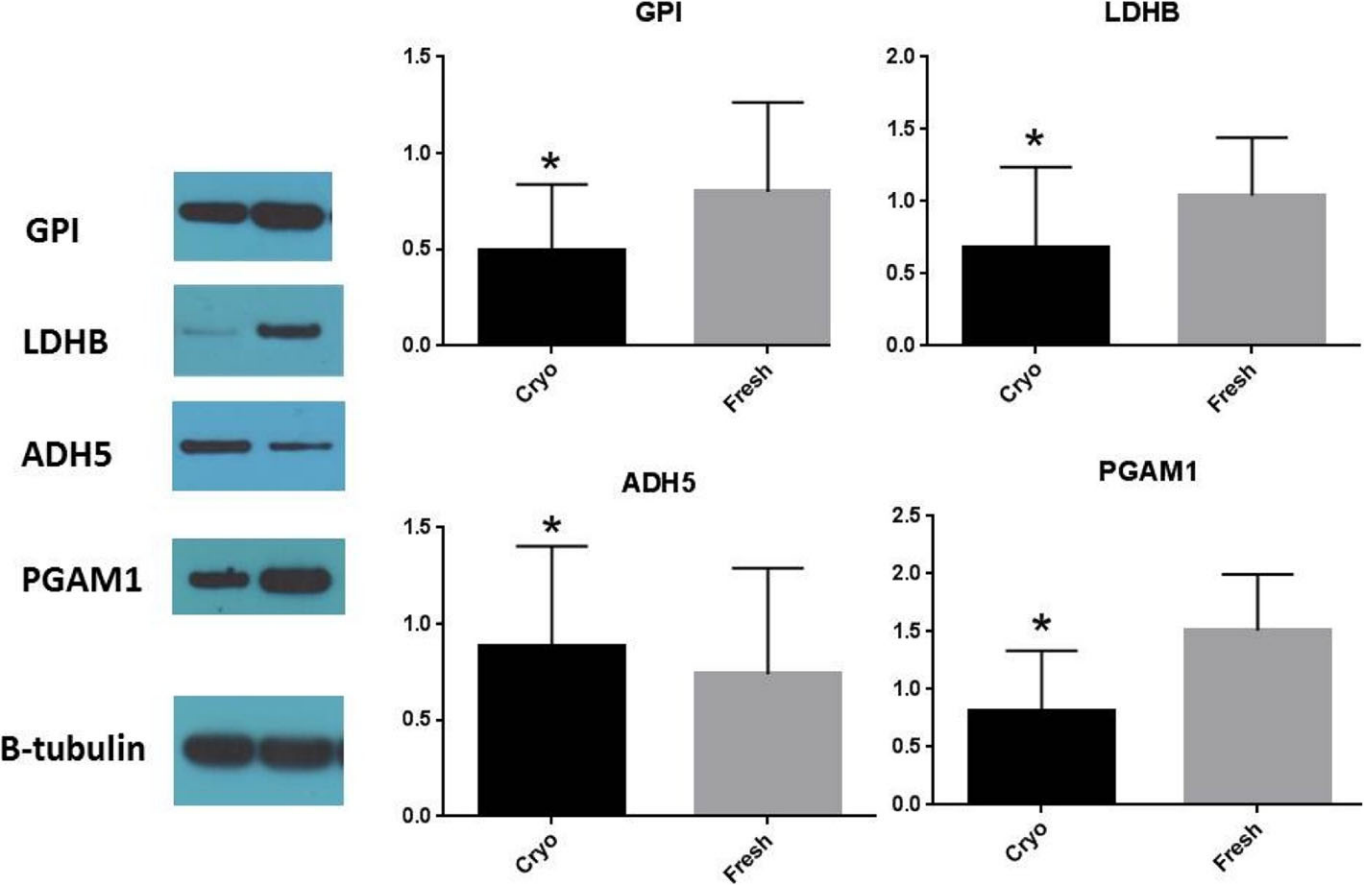
Validation of the 2DE results by Western blot

These protein analysis results confirmed the previous genomic analysis of metabolomics, and the results confirmed the differential protein levels observed via 2DE (Fig. 4). The cryopreserved group had lower levels of GPI, LDHB, and PGAM1. and a higher level of ADH5 than the fresh sperm (Fig. 4).

## Discussion

Worldwide, the percentage of male infertility ranges between 20 and 70% [20], and men with azoospermia or severe oligozoospermia will benefit from sperm cryopreservation. Furthermore, cryopreservation is a simple and effective technique for preserving fertility potential [4, 21]. However, after sperm cryopreservation, too many sperm lose their motility and fertility [5, 22]. Some sperm proteins have been recognized to be associated with sperm quality, and the loss of these proteins may be responsible for the decrease in fertility in sperm cryopreservation, such as: heat-shock protein 90 [23] and, Enolase1 (ENO1). However single protein bioresearch can only partly explain the cryodamage, and further study should be based on direct or indirect protein-protein interaction and mechanistic factors.

Proteomics technology has been identified as valuable tool for sperm [15, 24, 25]. Proteomic changes in human sperm as a result of cryoinjury have been reported previously. Wang et.al [13] found twenty-seven proteins that differed in abundance between fresh and cryopreserved sperm, by using two-dimensional polyacrylamide gel electrophoresis (2-DE) and mass spectrometry. However 2-DE has its limitations, including a low sensitivity of the densitometry analysis. Bogle et al. [26] used tandem mass tag (TMT) technology to identify potential proteomic changes at every stage of the cryopreservation process, but they did not compare fresh and cryopreserved sperm. Different from TMT, the DIA strategy has the characteristics of high quantitative accuracy and high reproducibility [11, 27]. Due to its global nature and enormous multiplexing capacity, DIA has been widely used in mechanistic studies and clinical biomarker screening in human reproduction for the enhanced protein coverage and analytical reproducibility [28]. In our study, a total of 174 significantly differential proteins were identified from 3790 quantitatively analyzed proteins, which is much more than previously reported discoveries [13, 26].

Improving upon previous studies on human cryopreserved sperm proteomics analysis [13, 26], our study performed KEGG analysis for the different proteins. KEGG is a frontier interdisciplinary subject based on life science and computer science. Bioinformatics analysis has been widely used as a powerful tool for data processing and prediction by employing various databases, and it has been widely used in the discovery of new biomarkers and the study of new therapeutic targets [29, 30]. According to the KEGG analysis, the present study revealed that the metabolic pathways were affected by cryopreservation. This result confirmed previous research. Wang et al. found the decreased abundance of succinyl-CoA:3-ketoacid CoA transferase (OXCT1) in cryopreservated sperm, and this enzyme is involved in ketone metabolism and might be associated with glycolysis in sperm [13]. According to Bogle et al. [26], energy- and metabolism- related proteins account for 15% of the collated differential proteins.

Different studies have proven that the pathway of carbon metabolism is associated with sperm and male infertility [31]. In addition, sperm are highly specialized mammalian cells; the sperm must reserve enough adenosine triphosphate (ATP) to maintain the physiological processes, including motility, capacitation, hyperactivation, acrosome reaction and fertilization, all of which are highly energy dependent processes. The ATP is formed via two metabolic pathways: glycolysis and oxidative phosphorylation (OXPHOS) [32], which are extremely down regulated in the post-thaw sperm. The present study is beneficial for the further study of energy metabolic pathways involved in cryoinjury.

While many studies have reported that glycolysis is the primary source of ATP during sperm motility [32–34], OXPHOS is involved in maturation and differentiation [35]. Many study have also shown that sperm motility will significantly decrease in the process of cryopreservation and that ATP is extremely decrease in the post-thaw sperm [5, 36, 37]. Based on thede results, we focuesd the glycolysis pathway in future research. The four proteins related to glycolysis were identified by Western boltting. This study shows the a correlation between GPI and sperm motility. Consistent with previous research [37], our study show that GPI is an important factor of thawed sperm. The variability of LDHB in the process of sperm cryopreservation has also been identified in the sturgeon [38]. Early research suggests that PGAM [39] and ADH [40] is associated with spermatogenic distinction and affects the function of cell proliferation, apoptosis and migration. Further research on the correlation between PGAM1/ADH and sperm cryopreservation requiresis needed.

## Conclusions

Human sperm cryopreservation is a simple and effective approach for male fertility preservation. To identify potential proteomic changes in this process, Data-independent acquisition (DIA), a proteomics technology with high quantitative accuracy and high reproducibility was used to quantitatively characterize the proteomics of human sperm cryopreservation. A total of 174 significantly differential proteins were identified between fresh and cryoperservated sperm: 98 were decreased and 76 were increased in the cryopreservation group. Bioinformatic analysis revealed that metabolic pathways play an important role in cryopreservation, including propanoate metabolism, glyoxylate and dicarboxylate metabolism, glycolysi/gluconeogenesis, and pyruvate metabolism. The four different proteins involved in glycolysis were identified by Western Blotting: GPI, LDHB, ADH5, and PGAM1. Our work will provide valuable information for future investigations and pathological studies involving sperm cryopreservation.

## Supplementary information

**Additional file 1: Table S1.** The differential proteins due to cryopreservtaion.

**Additional file 2: Table S2.** The results of the GO enrichment.

## Data Availability

The datasets for the current study are available from the corresponding author upon reasonable request.

## Abbreviations

BP: Biological process
CC: Cellular component
DDA: Data-dependent acquisition
DIA: Data-independent acquisition
FWHM: Full-Width Half-Maximum
MF: Molecular function
PPI: Protein-protein interaction
VAP: Average path velocity
VCL: Curvilinear velocity
VSL: Straight line velocity
